# Aryl Hydrocarbon Receptor (AhR) and Vascular Endothelial Growth Factor (VEGF) Crosstalk in Doxorubicin Nephrotoxicity: Mechanisms and Therapeutic Perspectives

**DOI:** 10.3390/cimb48010116

**Published:** 2026-01-22

**Authors:** Noha A. Alshuwayer, Qamraa H. Alqahtani, Marwa H. Hussein, Raeesa Mohammed, Iman H. Hasan

**Affiliations:** 1 Department of Anatomy, College of Medicine, King Saud University, P.O. Box 2925, Riyadh 11461, Saudi Arabia; nohamd@ksu.edu.sa (N.A.A.); rmohammad@ksu.edu.sa (R.M.); 2 Department of Pharmacology and Toxicology, College of Pharmacy, King Saud University, P.O. Box 22452, Riyadh 11495, Saudi Arabia; ghamad@ksu.edu.sa (Q.H.A.); mhussein3.c@ksu.edu.sa (M.H.H.)

**Keywords:** nanocurcumin, doxorubicin, VEGF, endothelial injury, nephrotoxicity

## Abstract

Doxorubicin (DOX), a widely used chemotherapeutic, is constrained by its nephrotoxicity, characterized by endothelial injury, inflammation, and oxidative stress. Vascular endothelial growth factor (VEGF) signaling in the kidney serves a dual function. Under normal conditions, it supports the survival of glomerular endothelial cells and maintains vascular stability, but when excessively activated, it disrupts angiogenesis and contributes to kidney injury. In this context, we hypothesize that Nanocurcumin (CUR-NP), a nano-formulated curcumin derivative with enhanced bioavailability, can modulate the VEGF pathway and restore regular renal activity. Thus, this study aims to explore the potential protective effect of CUR-NP on DOX-induced renal injury in male rats. Thirty-two Wistar albino rats were used and distributed into four groups. CUR-NP (80 mg/kg dissolved in 1% CMC) was administered by oral gavage for two weeks. A single dose of DOX (15 mg/kg) (i.p.) was injected on day seven of the study. Results showed that DOX increased the circulating creatinine, urea, and urea-nitrogen levels, while pretreatment with CUR-NP markedly alleviated kidney function. In addition, CUR-NP treatment significantly normalized oxidative stress markers in renal tissues, such as NO, GSH, and SOD, and improved renal pro-inflammatory mediators, TNF-α, IL-6, and NF-κB-p65. DOX caused degeneration of glomeruli and tubules with degenerated epithelial lining and casts in their lumens. Conversely, CUR-NP maintained standard tubular and glomerular structure. Immunohistochemistry showed that DOX strongly upregulated VEGF and AhR, while CUR-NP markedly reduced their expression, countering VEGF/AhR pathway disruption and helping restore physiological signaling.

## 1. Introduction

The aryl hydrocarbon receptor (AhR) is considered a ligand-activated transcription factor that binds to a wide range of small molecules and exogenous toxins. AhR is a critical regulator of many biological functions, such as cellular differentiation, immune response, and tumor formation [1]. In the kidney, AhR activation has been associated with endothelial dysfunction, inflammation, oxidative stress, and fibrosis [2]. Vascular endothelial growth factor (VEGF) is critical for maintaining vascular permeability, glomerular endothelial cell survival, and capillary integrity [3]. AhR induces VEGF expression through activating transcription factor 4 (ATF4) expression [4].

Sustained stimulation of the AhR by uremic toxins disrupts VEGF regulation, reducing vascular stability and leading to kidney damage. Additionally, AhR activation is linked to oxidative stress and increased NF-κB signaling, both of which affect VEGF levels in kidney endothelial and tubular epithelial cells. VEGF overexpression contributes to glomerular hyperpermeability, abnormal angiogenesis, and proteinuria [5].

Doxorubicin is a Quinone-containing anthracycline antibiotic that has been employed in the treatment of cancer since 1969 [6]. The use of DOX in chemotherapy has been limited mainly due to its cardiac, renal, pulmonary, testicular, and hematological toxicities [7]. The pathophysiological mechanisms underlying DOX-induced nephrotoxicity are multifactorial and not fully elucidated. Primary factors include depletion of antioxidant defenses (e.g., SOD) with oxidative stress, lipid peroxidation, tubulointerstitial inflammation, and cytokine surges such as tumor necrosis factor-alpha (TNF-α), interleukin-1beta (IL-1β), and apoptosis, which eventually lead to tubular fibrosis and atrophy with significant proteinuria and hematuria [8]. Experimental work has shown that DOX induces AhR nuclear translocation and increases expression of AhR-modulated genes in vitro, indicating that DOX is able to engage the AhR pathway [9]. DOX also modulates VEGF by other routes, as it produces ROS and mitochondrial damage [10]. In turn, these effects can modulate hypoxia-inducible factor 1-alpha (HIF-1α) stability and signaling [11,12] and lead to dysregulation of miRNAs (e.g., miR-320a) [13].

Curcumin, a naturally occurring polyphenolic compound extracted from *Curcuma longa*, demonstrates potent anti-inflammatory, antioxidant, and anti-apoptotic properties. Despite its promising pharmacological profile, curcumin’s clinical utility is significantly hindered by its poor bioavailability. Recent progress in nanotechnology has enabled the creation of nanocurcumin (NCR-NP) formulations that significantly boost their solubility, absorption by cells, and distribution throughout tissues. Nano-formulations have been shown to enhance water solubility and cellular uptake, thereby improving tissue distribution and overall bioavailability. Liposomal formulations, consisting of phospholipid vesicles, not only increase the solubility of curcumin but also protect it from gastrointestinal degradation [14]. Evidence suggests that liposomal NCR prolongs its circulation time and significantly augments plasma bioavailability [15]. In addition, liposomes facilitate intracellular delivery through mechanisms such as membrane fusion or endocytosis, thereby enhancing the uptake of encapsulated curcumin. This process contributes to greater therapeutic efficacy, even at lower dosages [16].

These nanoscale systems not only improve curcumin’s bioavailability but also support controlled drug release, extending its therapeutic effects and enhancing its overall potency [17].

CUR-NP has been shown to modulate VEGF expression in cancer and ocular cells [18]. In hepatocellular carcinoma, downregulation of VEGF occurs following exposure of the tumor cells to curcumin- and DOX-loaded lipid nanoparticles [19,20]. However, there is limited in vivo research addressing the effects of CUR-NP on VEGF in the context of DOX-induced renal injury. Furthermore, CUR-NP activates the Nrf2/HO-1 pathway while inhibiting NF-κB and proinflammatory cytokines; by reducing ROS and inflammation, CUR-NP could normalize stress-induced VEGF dysregulation. Our recent studies showed that CUR-NP activated Nrf2/HO-1 in copper-induced lung injury, suppressing oxidative damage and inflammation [21]. In addition, Xinpu Chen et al., 2014 found that CUR-NP reduced NF-κB activity by 35% and downregulated pro-inflammatory cytokines like IL-6, IL-8, and TNF-α [22]. Moreover, M. Lebda et al., 2022 further confirmed these effects, showing CUR-NP significantly attenuated pro-inflammatory cytokines (TNF-α and IL-1β) while improving Nrf2 activity [23].

The evidence spans diverse models including lung injury, mastitis, and cellular inflammation, suggesting a robust effect. We hypothesized that this is the predominant CUR-NP reno-protective rationale.

In summary, the protective effects of CUR-NP in DOX nephrotoxicity likely involve antioxidant defense through upregulation of Nrf2/HO-1 and restoration of superoxide dismutase (SOD) and glutathione (GSH) activity. In addition, CUR-NP displays anti-inflammatory properties by suppressing NF-κB signaling and lowering concentrations of key pro-inflammatory cytokines, including TNF-α and IL-6, suggesting a role in alleviating renal inflammation. Furthermore, CUR-NP protects the glomerular and tubular architecture. Given that CUR-NP activates Nrf2 and influences related pathways, this indicates a possible mechanism for the modulation of AhR-VEGF pathways by CUR-NP and may enhance the preservation of renal microvascular health following DOX exposure. Targeting AhR signaling and fine-tuning VEGF activity, therefore, represent promising therapeutic strategies to mitigate chemotherapy-induced nephrotoxicity while preserving renal microvascular health. This makes CUR-NP a promising candidate for mitigating DOX nephrotoxicity.

## 2. Materials and Methods

### 2.1. Chemicals

Doxorubicin was acquired from Sigma (St. Louis, MO, USA), while CUR-NP was supplied by Lipolife (Coggeshall, UK) (The product contains patented C3^®^ Curcumin, providing 95% curcuminoids per dose, encapsulated within lipolife^®^ liposomes to optimize absorption), Kidney function assays (Creatinine, urea, and uric acid) were acquired from Randox Company (London, UK). ELISA kits for TNF-α (SEKR-0009) and IL-6 (SERK-0005) were provided by Solarbio Life Sciences Company (Beijing, China), while ELISA kits for NF-κB-p65 (NBP2-29661-1) were supplied by Novus Biologicals (Centennial, CO, USA). Primary antibodies against VEGF (cat. ab32152) and AhR (cat. MA1-514) were provided by Abcam (Centennial, CO, USA) and Thermo Fisher (Invitrogen) (Dartford, UK), respectively, and β-actin (Cat. NO, 4967) was provided by Cell Signaling, Danvers, MA, USA. Other lab chemicals used in biochemical and histopathological examination were acquired from Sigma (St. Louis, MO, USA).

### 2.2. Animals and Experimental Design

**Figure 1:**
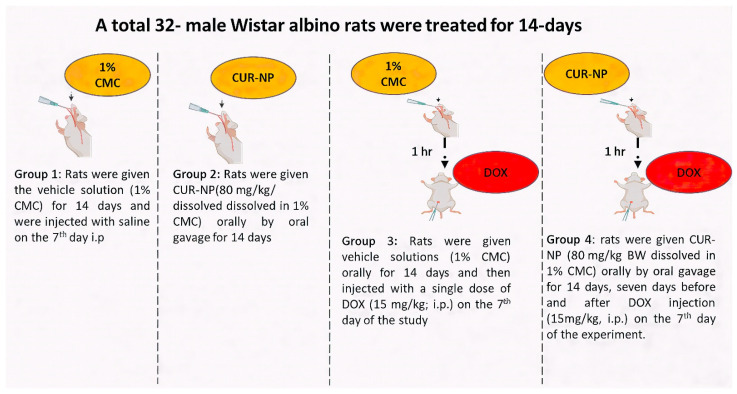
Expermintal animal design. A schematic illustration of the experimental design and treatment protocol used in the animal study. The dose of curcumin nanoparticles (CUR-NP) was selected based on our previously published work [24], while the doxorubicin (DOX) dose was chosen according to established protocols reported in the literature [25].

Adult male Wistar albino rats (150–200 g) were collected from the Bio-Resource Unit at the College of Pharmacy, King Saud University, Saudi Arabia. Thirty-two rats were randomly grouped and housed at a standard humidity (25 °C) with a 12 h light/dark cycle. They had free access to food and water. The experimental design (Figure 1) was established in accordance with the recommendations of the Institutional Animal Care and approved by the Research Ethics Committee at King Saud University (KSU-SE-24-68).

On day 15, the rats were fasted for 12 h, then weighed and anesthetized using carbon dioxide (CO_2_), sacrificed by decapitation, and blood and kidney samples were collected. Serum samples were collected after blood centrifugation at 3000 rpm at 4 °C for 30 min and then stored at −80 °C. The kidney tissues were carefully separated and washed in cold phosphate-buffered saline (PBS) and divided into three parts. The first part was fixed in 10% formalin solution, while the second part was homogenized (10% *w*/*v*) in Tris-HCl buffer (pH 7.4). Centrifugation of the homogenate and collection of the supernatant were carried out so that they could be used for the determination of antioxidant and inflammatory biomarkers. The last part of the tissue samples was cryopreserved at −80 °C for RT-PCR gene expression analysis.

### 2.3. Serum Creatinine, Urea, and Urea Nitrogen

After the preparation of serum samples, the serum levels of creatinine, urea, and urea nitrogen were evaluated using a colorimetric assay following the manufacturer’s directions.

### 2.4. Assay of Oxidative Stress and Inflammatory Mediators

Renal Reduced glutathione (GSH) level was determined using a commercial kit according to the previously established Ellman’s method (1959) (Thermo Fisher). Following Beutler et al. [26]. Catalase activity in kidney tissue was evaluated using a colorimetric kit (EIACATC, Thermo Fisher Co.). Superoxide dismutase (SOD) activity was estimated by applying the Marklund 1974 method [27]. Finally, the renal NO level following the Grisham et al. (1996) method [28]. Levels of renal NF-κB-p65 and the pro-inflammatory biomarkers TNF-α and IL-6 were determined using ELISA kits according to the manufacturer’s instructions.

### 2.5. Western Blotting and Protein Expression Analysis

Western blotting was employed to evaluate protein expression levels in kidney tissues, as previously described. Briefly, proteins were extracted and quantified according to the method reported in previous research [29]. Equal amounts of protein (40 μg) were separated on 12% SDS-PAGE gels and subsequently transferred onto PVDF membranes. After blocking with 5% non-fat dry milk for 1 h at room temperature, the membranes were incubated overnight at 4 °C in PBST buffer with primary antibodies against VEGF and AhR (1:100), along with β-actin (1:1000) as a loading control. Following washing, the membranes were incubated with HRP-conjugated secondary antibodies (1:2000) for 1 h at room temperature. Protein bands were then visualized using an ECL detection system, captured using the Image Quant LAS 4000 imaging platform (Cytiva, Marlborough, MA, USA), and quantified relative to β-actin expression.

### 2.6. Histopathological and Immunohistochemistry (IHC) Examination

Directly after the rats were sacrificed, three kidney tissues from the control and all the treated groups were fixed in 10% formaldehyde for one day. The fixed tissues were processed to prepare 5-μm-thick paraffin sections for staining with hematoxylin and eosin (H&E) to examine the kidney’s structure or with Masson’s Trichrome stain to detect fibrosis in tissues. Other kidney sections were processed for the immunohistochemical detection of VEGF and AhR. Briefly, the tissue sections mounted on slides were blocked by immersion in 3% hydrogen peroxide (H_2_O_2_) solution for 5 min. After washing in Tris-buffered saline (TBS; pH 7.6) for 10 min, the slides were incubated with protein block (Newcastle, UK) for 5 min to block the non-specific binding of antibodies. The sections were probed with diluted primary polyclonal antibody for VEGF (1:50) and AhR (1:100) overnight at 4 °C. On the second day, the slides were incubated with a protein blocking solution. After washing with phosphate-buffered saline (PBS), slides were incubated with the secondary antibody for 60 min at 25 °C. Color development was achieved using a 3,3′-Diaminobenzidine (DAB) detection kit. Image analysis of immuno-stained sections was performed using ImageJ software (version 1.32j, National Institutes of Health) (NIH, Bethesda, MD, USA), and data were expressed as a percentage relative to the control group.

### 2.7. Statistical Analysis

The findings are expressed as the mean ± standard error of the mean (SEM). One-way ANOVA was used, followed by Tukey post hoc test for multiple comparison after applying the Shapiro–Wilk test and confirming the homogeneity of variances using Levene’s test. GraphPad Prism 9 was used for statistical analysis. The data were considered statistically significant if the *p*-value < 0.05.

## 3. Results

### 3.1. CUR-NP Attenuates DOX-Induced Renal Hypertrophy

**Figure 2:**
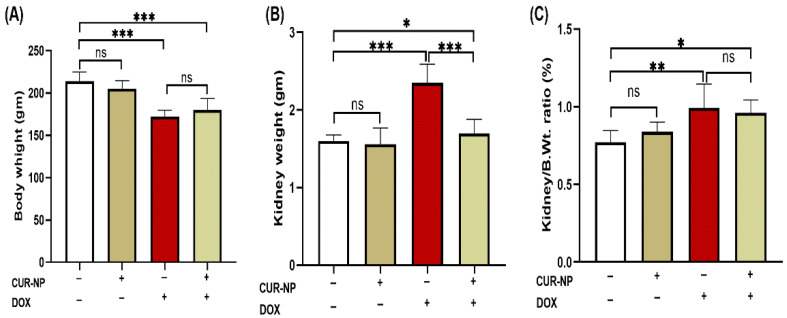
Effects of curcumin nanoparticles (CUR-NP) on body and kidney weight parameters in doxorubicin (DOX)-induced nephrotoxicity. CUR-NP treatment did not alter body weight loss (**A**), significantly reduced kidney weight increase (**B**), and had no effect on the kidney weight-to-body weight ratio (**C**) in DOX-treated rats. Data are presented as mean ± SEM (*n* = 8). Statistical significance is indicated as follows: ns, not significant; * *p* < 0.05; ** *p* < 0.01; *** *p* < 0.001. DOX, doxorubicin; CUR-NP, curcumin nanoparticles.

DOX-administered rats showed a significant (*p* < 0.001) decline in body weight and increase in kidney weight at the end of the experiment when compared with the control rats (Figure 2A,B). Treatment with CUR-NP significantly (*p* < 0.01) prevented the kidney weight increase in DOX-administered rats, while it did not affect the body weight of treated rats. The relative kidney weight (kidney weight/body weight ratio) of normal rats treated with CUR-N was not affected as compared with the control group. DOX-administered rats showed significantly (*p* < 0.001) increased kidney weight/body weight ratio, which was not altered by CUR-NP treatment as compared to DOX-administered rats (Figure 2C).

### 3.2. CUR-NP Reduces Altered Kidney Function and Oxidative Stress Caused by DOX-Induced Nephrotoxicity

**Figure 3:**
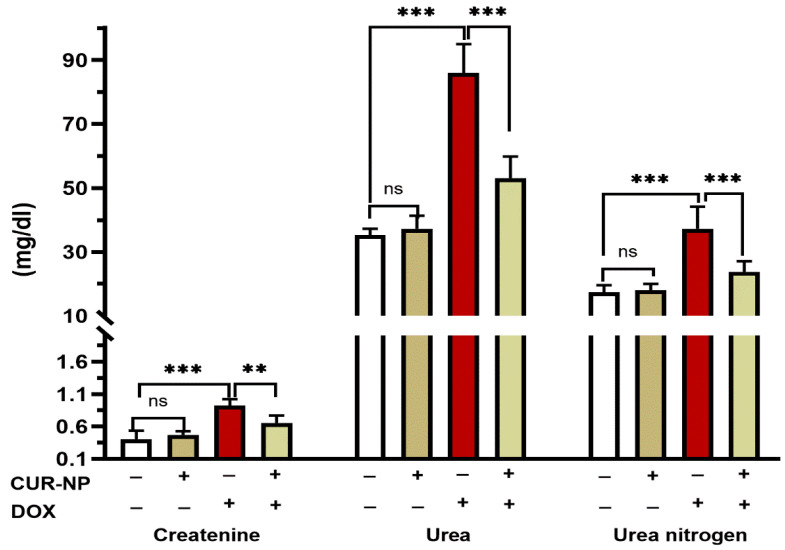
CUR-NP improves kidney function markers in DOX-induced nephrotoxicity in rats. Data are expressed as mean ± SEM (*n* = 8). (ns; non significant, ** *p* < 0.01, *** *p* < 0.001). DOX, doxorubicin, CUR-NP, curcumin-nanoparticle.

DOX- administrated rats showed a significant (*p* < 0.001) increase in the circulating levels of creatinine, urea, and urea nitrogen when compared with the control rats (Figure 3). Pretreatment of rats with CUR-NP markedly alleviated the increase in the levels of serum creatinine (*p* < 0.001), urea (*p* < 0.001), and urea nitrogen (*p* < 0.01). Rats receiving CUR-NP only showed non-significant changes in kidney function markers when compared with the control.

The impact of CUR-NP on the redox status of rats was evaluated through the assessment of the levels of NO and antioxidants. When compared with the control rats, rats that received CUR-NP showed no significant changes in renal NO, GSH, SOD, or Catalase (CAT) levels (Table 1). On the other hand, DOX-administered rats exhibited a significant increase in kidney NO (*p* < 0.001) level. Treatment with CUR-NP significantly decreased renal NO (*p* < 0.001) levels. The kidney of DOX-administered rats showed significantly declined renal level of GSH (*p* < 0.001) and the activities of both SOD (*p* < 0.001) and CAT (*p* < 0.05). CUR-NP markedly alleviated the decrease in GSH levels (*p* < 0.05) and SOD activity (*p* < 0.01), while catalase activity remained unchanged (Table 1).

### 3.3. CUR-NP Mitigates Kidney Damage in DOX-Administered Rats

**Figure 4:**
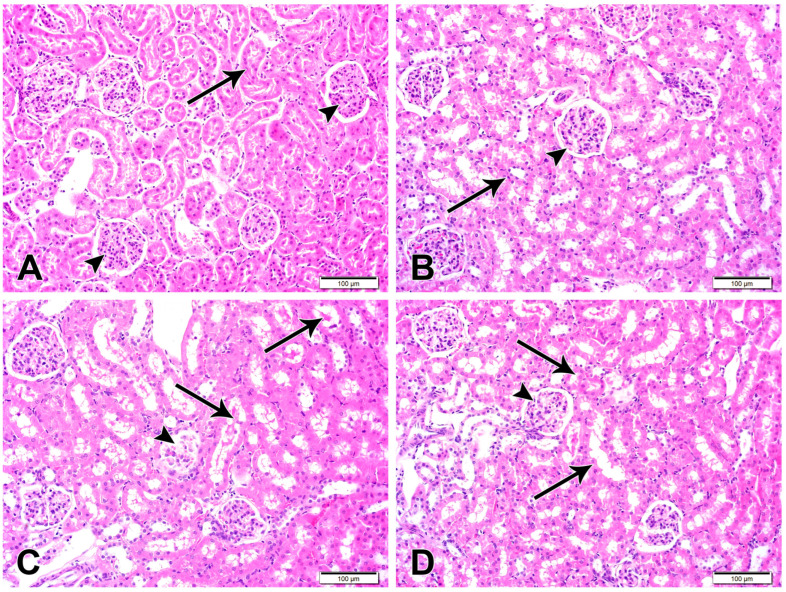
Representative kidney sections are shown (scale bar = 100 µm). (**A**) Kidney section from a normal control rat exhibiting normal renal cortical architecture with intact renal corpuscles (arrowheads) and renal tubules (arrow). (**B**) Kidney section from a CUR-NP–treated rat showing preserved renal corpuscles (arrowhead) and tubular structures (arrow) comparable to the control group. (**C**) Kidney section from a doxorubicin (DOX)-treated rat demonstrating extensive degeneration of renal corpuscles (arrowhead) and tubular damage characterized by luminal casts and degenerated epithelial lining (arrows). (**D**) Kidney section from a rat treated with DOX and CUR-NP showing a marked attenuation of DOX-induced pathological alterations in both renal corpuscles (arrowhead) and renal tubules (arrows).

Histological examination of the H&E-stained kidney sections of control (Figure 4) and CUR-NP-treated rats revealed a typical structure of the kidney with normal glomeruli and renal tubules. Sections in the kidney of DOX-administered rats showed several histological alterations, including enlargement of renal corpuscles and narrowing of the renal spaces due to the glomerular hypercellularity (Figure 4). DOX-intoxicated rats treated with CUR-NP showed an improvement in the structure of the kidney, as shown in Figure 4.

### 3.4. CUR-NP Mitigates Inflammatory Biomarkers and NF-κB-P65 in DOX-Administered Rats

**Figure 5:**
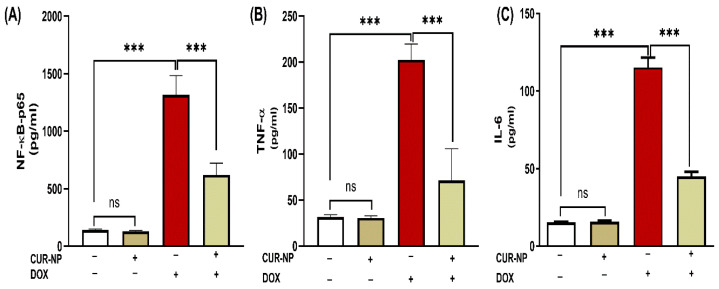
CUR-NP improves renal pro-inflammatory mediators (**A**) NF-κB-P65, (**B**) TNF-α, and (**C**) IL-6 in DOX-induced nephrotoxicity in rats. Data are expressed as mean ± SEM (*n* = 8). (ns; non significant, *** *p* < 0.001). DOX, doxorubicin, CUR-NP, curcumin-nanoparticle.

The effect of CUR-NP on the inflammatory response in rats’ DOX-induced kidney toxicity was evaluated via assessment of changes in NF-κB-p65 and pro-inflammatory mediators. DOX-induced toxicity was manifested in elevated NF-κB-p65 levels (Figure 5A) along with increased kidney TNF-α (Figure 5B) and IL-6 (Figure 5C) (*p* < 0.001). Treatment with CUR-NP effectively decreased NF-κB-p65 and pro-inflammatory mediators (TNF-α and IL-6) in renal tissues.

### 3.5. CUR-NP Ameliorates the Increased Protein Expression of AhR and VEGF in DOX-Induced Nephrotoxicity

**Figure 6:**
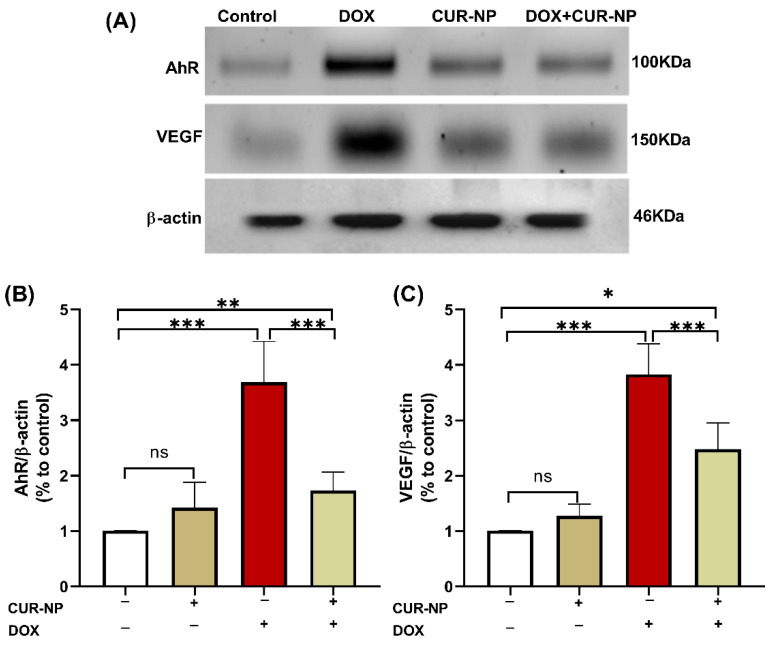
Effect of CUR-NP on renal AhR and VEGF expression. (**A**) Representative Western blotting of AhR, VEGF, and β-actin. (**B**,**C**) CUR-NP down-regulated renal AhR and VEGF, respectively. Data are expressed as mean ± SEM (ns; non-significant, * *p* < 0.05, ** *p* < 0.01, *** *p* < 0.001). DOX, doxorubicin, CUR-NP, curcumin-nanoparticle.

**Figure 7:**
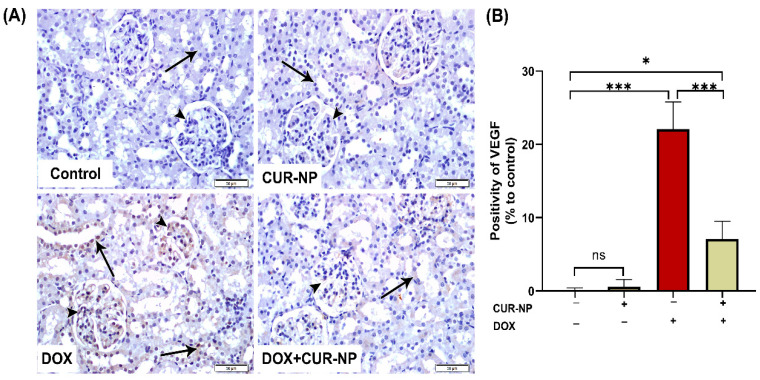
Vascular endothelial growth factor (VEGF) immunohistochemical expression in renal tissue. (**A**) Representative VEGF-immuno-stained kidney sections (scale bar = 50 µm). (Control) Kidney section from a normal control rat showing absence of VEGF immunoreactivity in the epithelial cells of renal corpuscles (arrowheads) and renal tubules (arrow). (CUR-NP) Kidney section from a CUR-NP–treated rat exhibiting similarly negative VEGF immunoreactivity in renal corpuscles (arrowhead) and tubules (arrow). (DOX) Kidney section from a doxorubicin (DOX)-treated rat demonstrating marked VEGF immunopositivity in renal corpuscles (arrowhead) and renal tubules (arrows). (DOX + CUR-NP) Kidney section from a rat treated with DOX and CUR-NP showing a pronounced reduction in VEGF immunoreactivity in both renal corpuscles (arrowhead) and tubules (arrows). (**B**) Quantitative analysis of VEGF-immuno-positive cells in renal tissue. Data are expressed as mean ± SEM. Statistical significance is indicated as follows: ns, not significant; * *p* < 0.05; *** *p* < 0.001. DOX, doxorubicin; CUR-NP, curcumin nanoparticles.

**Figure 8:**
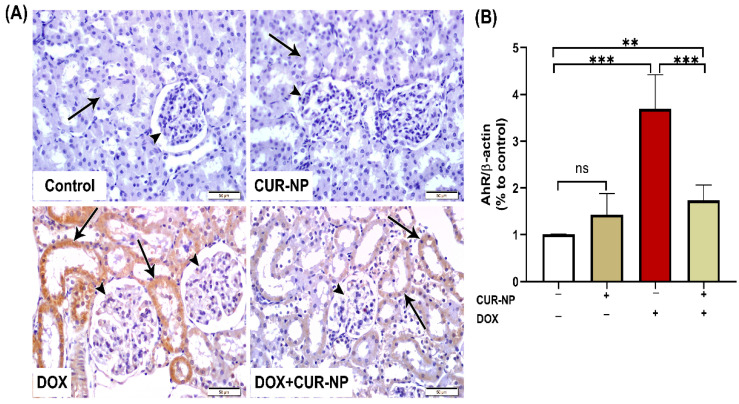
Aryl hydrocarbon receptor (AhR) immunohistochemical expression in renal tissue. (**A**) Representative AhR-immuno-stained kidney sections (scale bar = 50 µm). (Control) Kidney section from a normal control rat showing absence of AhR immunoreactivity in renal corpuscles (arrowheads) and renal tubules (arrow). (CUR-NP) Kidney section from a CUR-NP–treated rat exhibiting similarly negative AhR immunoreactivity in renal corpuscles (arrowhead) and renal tubules (arrow). (DOX) Kidney section from a doxorubicin (DOX)-treated rat showing marked AhR immunopositivity in tubular epithelial cells lining the renal tubules (arrows), while renal corpuscle cells remain unstained (arrowheads). (DOX + CUR-NP) Kidney section from a rat treated with DOX and CUR-NP demonstrating a pronounced reduction in AhR immunoreactivity in renal tubular cells (arrows), with renal corpuscle cells remaining negative (arrowheads). (**B**) Quantitative analysis of AhR-immunopositive reactions in renal tissue. Data are expressed as mean ± SEM. Statistical significance is indicated as follows: ** *p* < 0.01; *** *p* < 0.001. DOX, doxorubicin; CUR-NP, curcumin nanoparticles.

In nephrotoxicity, activation of the AhR contributes to renal injury by promoting oxidative stress and inflammation, enhancing the generation of reactive oxygen species (ROS), inducing pro-inflammatory cytokine expression, and stimulating extracellular matrix deposition. Dysregulation of AhR signaling has been strongly implicated in the progression of drug-induced nephrotoxicity, as well as in endothelial dysfunction and vascular injury within the renal microcirculation. Persistent AhR activation further exacerbates tubular injury, podocyte damage, and glomerular dysfunction. Protein expression was estimated by Western blot (Figure 6), and immunohistochemical (IHC) and quantitative analysis for protein expression of VEGF (Figure 7) and AhR expression Western blot analysis (Figure 8) revealed a significant upregulation of proteins in the DOX-treated group compared with control rats. In contrast, CUR-NP treatment markedly downregulated VEGF and AhR expression. 

## 4. Discussion

The current study demonstrates the therapeutic potential of CUR-NP in alleviating DOX-induced renal toxicity through a multifaceted approach. CUR-NP appears to exert protective effects by targeting several key pathological mechanisms underlying DOX nephrotoxicity. These include the suppression of oxidative stress, modulation of inflammatory signaling pathways, preservation of endothelial integrity, and correction of microvascular dysfunction. Collectively, these actions contribute to the attenuation of structural damage within both glomerular and tubular structures. Conventional curcumin has shown protective effects in other experimental DOX organs, but its clinical translation is limited by its poor solubility and bioavailability. Nano formulations have been designed to overcome these challenges and may enhance therapeutic efficacy.

According to our results, CUR-NP was able to inhibit the AhR-VEGF signaling cascade and downregulate NF-κB-p65 pathway activation. Additionally, it effectively reduces lipid peroxidation and restores the activity of intrinsic antioxidant enzymes. These molecular actions contribute to the renewal of normal architecture in the kidney’s glomerular and tubular structures. Collectively, these actions directly address the molecular pathways involved in DOX nephrotoxicity. Our study results have shown that DOX induced a significant reduction in body weight, with increased kidney weight and kidney/body weight. Ratio which align with previous literature [30]. In contrast, CUR-NP repressed this effect and reduced renal tissue damage.

In our study, DOX-induced renal toxicity could be partly mediated by reduced activities of antioxidant enzymes such as SOD and CAT, with GSH depletion and increased NO, which confirms the induction of oxidative stress. These results support previous results of [31], who found that DOX induced a remarkable diminution in renal SOD level, accompanied by a decrement of GSH content. However, CUR-NP was able to restore the antioxidant enzyme activities in kidney tissues significantly. Similarly, these findings agree with the previous study of [32].

In addition to oxidative stress, DOX induced inflammation through the upregulation of the expression of pro-inflammatory markers, TNF-α and IL-6, in addition to activation of the NF-κβ inflammatory signaling pathway. These findings are consistent with a previous study of DOX effects on rat kidneys conducted by [33]. The observed inflammatory response can be mechanistically attributed to oxidative stress, which can induce inflammation through activation of redox-sensitive transcription factor activation, notably NF-kB, thereby regulating the expression of several pro-inflammatory cytokines [33]. However, CUR-NP co-treatment effectively reduced TNF-α and IL-6 and suppressed NF-κβ-p65 signaling. It was suggested that CUR-NP-induced anti-inflammatory effect against DOX-induced nephrotoxicity could be attributed to the upregulation of Peroxisome proliferator-activated receptor gamma (PPAP-γ), which is involved in anti-inflammatory reactions via TNF-α downregulation [34]. These findings are consistent with previous studies by [35,36], respectively.

DOX-induced oxidative stress and inflammation may compromise the structural stability of kidney cells, potentially resulting in renal impairment characterized by elevated serum levels of BUN, urea, and creatinine, along with marked histopathological alterations [30,37]. Conversely, CUR-NP improved the blood urea, BUN, and creatinine levels and improved kidney histology. These results are consistent with a previous study of [38].

The observed glomerular damage and cast formation in this study appear to stem from oxidative stress triggered by an overproduction of free radicals, reactive species, and inflammatory responses. Administration of CUR-NP mitigates these effects, evidenced by decreasing tubular lumen casts, glomerular shrinkage, and glomerulosclerosis. These histological improvements are attributed to the potent antioxidant and anti-inflammatory actions of CUR-NP and its nanoparticle formulation.

In the context of nephrotoxicity, activation of the AhR has been identified as a pivotal contributor to renal pathology. This activation facilitates oxidative stress and inflammatory cascades by augmenting the production of ROS, upregulating pro-inflammatory cytokines, and promoting extracellular matrix accumulation. Multiple studies provide strong evidence that AhR activation triggers oxidative stress and inflammatory responses in renal tissues [39]. Specifically, AhR activation augments ROS production, with C. Vogel et al., 2020 demonstrating that this process can lead to excessive ROS generation [40].

Mechanistically, AhR facilitates inflammatory cascades by upregulating pro-inflammatory cytokines and promoting extracellular matrix accumulation. In diabetic nephropathy models, AhR knockout significantly attenuated mesangial cell activation, macrophage infiltration, and extracellular matrix accumulation [41].

The evidence suggests AhR is not just a passive receptor but an active mediator of renal pathology, with multiple converging mechanisms of tissue damage. Aberrant AhR signaling is strongly associated with the progression of drug-induced renal injury, as well as with endothelial dysfunction and microvascular damage within the renal architecture. Multiple studies provide robust evidence for this association. Curran et al., 2022 demonstrated that AhR activation can either promote or protect against kidney injury depending on specific ligands [42]. In addition, S. Ito et al., 2016 specifically showed that AhR mediates vascular inflammation through enhanced leukocyte-endothelial interactions [42]. Furthermore, Cindy Nguyen et al., 2022 confirmed AhR’s role in endothelial dysfunction, revealing that AhR activation increases oxidative stress and disrupts vascular function [43].

The mechanism involves AhR’s ability to regulate critical cellular processes, including cell proliferation, inflammation, and oxidative stress [44]. Notably, Hongyan Xie et al., 2024 emphasize that AhR recognizes multiple toxins that can accumulate during kidney disease, making it a crucial mediator of renal pathology [45]. Sustained AHR activation further intensifies tubular epithelial degeneration, podocyte disruption, and glomerular impairment, thereby exacerbating overall renal dysfunction [46].

We propose that CUR-NP influences the AhR–VEGF axis through both direct and indirect mechanisms. The predominant protective effect in the kidney appears to be mediated via its antioxidant and anti-inflammatory properties, which attenuate HIF-1α stabilization and the inflammation-driven induction of VEGF, and consequently reduce the generation of endogenous AhR agonists. Collectively, these actions result in diminished VEGF expression and decreased pathological vascular leakage. AhR activity may therefore decline as a secondary effect, indicating that the AhR–VEGF interaction is modulated indirectly rather than through direct CUR–AhR binding [47].

An alternative mechanism involves direct AhR antagonism/agonism. CUR-NP may function as a partial AhR ligand or antagonist, thereby modulating canonical AhR-dependent transcription and subsequently influencing VEGF expression. This pathway is expected to generate a rapid canonical AhR transcriptional response, such as the induction or suppression of CYP1A1 [48].

Western blot analysis revealed that DOX administration elicited a marked upregulation of AhR and VEGF protein expression in renal tissue. This observation is consistent with prior reports demonstrating that DOX-induced oxidative stress and inflammatory signaling activate AhR [9], which subsequently contributes to dysregulated angiogenic and vascular responses, including enhanced VEGF expression. Aberrant activation of the AhR–VEGF signaling axis has been implicated in glomerular and tubular injury, endothelial dysfunction, and increased vascular permeability [44] hallmark features of DOX-induced nephrotoxicity.

In contrast, in our study, treatment with CUR-NP resulted in a pronounced downregulation of renal AhR and VEGF expression, paralleling significant improvements in biochemical indices, histopathological alterations, and renal functional outcomes. The suppression of AhR and VEGF by CUR-NP likely reflects a combination of direct modulation of AhR-dependent pathways and indirect effects mediated through curcumin’s potent antioxidant and anti-inflammatory properties, both of which are known to attenuate AhR activation. Notably, Nano formulation enhances curcumin’s bioavailability and tissue penetration, thereby augmenting its capacity to regulate redox-sensitive signaling cascades implicated in renal injury.

In the current study, immunohistochemical analysis of VEGF and AhR protein expression revealed a significant upregulation due to DOX-treatment, which agrees with a previous study of [9]. On AhR, despite being protective in cardiac tissue [49] indicates that: to improve cardiovascular Dox-toxicity, it is possible to prescribe anti-VEGF/VEGFR as it is upregulated due to inflammation and oxidative stress cascades. On the other hand, CUR-NP treatment markedly downregulated VEGF and AhR expression. These results agree with the previous study of [50], who found that CUR-NP inhibits angiogenesis in zebrafish by downregulating the HIF1A/VEGFA signaling pathway, which reveals the key role of nanocurcumin in angiogenesis, in addition to the previous study of [50] on endosomal CUR-NP. They found that CUR-NP is involved in angiogenesis by reducing the expression of the VEGF gene in Burkitt’s lymphoma. Moreover, CUR-NP-induced downregulation of AhR agrees with a previous study of [51], who found that curcumin suppresses AhR transformation by inhibiting its phosphorylation. Fortunately, several lines of evidence showed the absence of antagonistic pharmacodynamic effects. First, recent comprehensive analyses of 179 research studies, including clinical trials and animal and in vitro studies, demonstrated that antioxidant supplementation, including curcumin, with chemotherapy results in greater anticancer efficiency and prolongation in survival times in patients [52]. Second, curcumin exerts synergistic cytotoxic effects with doxorubicin in cell line models of cancer through modulation of distinct molecular pathways (like NF-kB, Nrf2, and caspase-3 activation) rather than competing for the same targets [53].

Collectively, these findings indicate that attenuation of DOX-induced AhR and VEGF overexpression constitutes an important mechanistic component of the renoprotective actions of CUR-NP, while acknowledging that additional molecular pathways are likely to contribute to its overall protective profile.

## 5. Conclusions

Nanocurcumin represents a promising adjunctive strategy to mitigate DOX-induced renal injury. It acts through multiple, complementary mechanisms that directly address key pathophysiologic processes of DOX nephrotoxicity.

Nanocurcumin exerts potent antioxidant and anti-inflammatory effects by suppressing NF-κB-p65, thereby indirectly regulating VEGF expression. In addition, CUR-NP downregulates VEGF and AHR expression and interferes with angiogenic signaling, which in turn attenuates tubular epithelial degeneration and glomerular impairment, thereby improving overall renal function and preserving normal architecture.

## Figures and Tables

**Table 1:** Effect of CUR-NP on oxidative stress biomarkers in control and DOX-administered rats.

Parameters	NO (μ mol/mg Tissue)	GSH (nmol/mg Tissue)	SOD (U/mg Protein)	Catalase (U/g)
Groups				
Control	2.5 ± 0.06	22.7 ± 0.7	19.6 ± 0.3	3.71 ± 0.23
CUR-NP	2.33 ± 0.2	21.8 ± 0.6	19.1 ± 0.7	3.9 ± 0.22
DOX	3.4 ± 0.2 **	15.6 ± 0.7 ***	10.5 ± 0.7 ***	2.68 ± 0.27 *
DOX + CUR-NP	2.66 ± 0.2 ^#^	18.6 ± 1.2 ^#^	14.3 ± 0.9 ^##^	3.1 ± 0.12 ^ns^

Data are expressed as mean ± SEM, *n* = 8. (* *p* < 0.05, ** *p* < 0.01, *** *p* < 0.001 versus Control and ^#^ *p* < 0.05, ^##^ *p* < 0.01, versus DOX-group). DOX, doxorubicin, CUR-NP, curcumin-nanoparticle, ns, nonsignificant.

## Data Availability

The original contributions presented in this study are included in the article. Further inquiries can be directed to the corresponding author.
